# Vision Impairs the Abilities of Bats to Avoid Colliding with Stationary Obstacles

**DOI:** 10.1371/journal.pone.0013912

**Published:** 2010-11-09

**Authors:** Dara N. Orbach, Brock Fenton

**Affiliations:** Department of Biology, University of Western Ontario, London, Ontario, Canada; University of Bern, Switzerland

## Abstract

**Background:**

Free-flying insectivorous bats occasionally collide with stationary objects they should easily detect by echolocation and avoid. Collisions often occur with lighted objects, suggesting ambient light may deleteriously affect obstacle avoidance capabilities. We tested the hypothesis that free-flying bats may orient by vision when they collide with some obstacles. We additionally tested whether acoustic distractions, such as “distress calls” of other bats, contributed to probabilities of collision.

**Methodology/Principal Findings:**

To investigate the role of visual cues in the collisions of free-flying little brown bats (*Myotis lucifugus*) with stationary objects, we set up obstacles in an area of high bat traffic during swarming. We used combinations of light intensities and visually dissimilar obstacles to verify that bats orient by vision. In early August, bats collided more often in the light than the dark, and probabilities of collision varied with the visibility of obstacles. However, the probabilities of collisions altered in mid to late August, coincident with the start of behavioural, hormonal, and physiological changes occurring during swarming and mating. Distress calls did not distract bats and increase the incidence of collisions.

**Conclusions/Significance:**

Our findings indicate that visual cues are more important for free-flying bats than previously recognized, suggesting integration of multi-sensory modalities during orientation. Furthermore, our study highlights differences between responses of captive and wild bats, indicating a need for more field experiments.

## Introduction

Many anecdotal reports describe bats colliding with large stationary objects such as television towers [1]–[6], lighthouses [7], and windows [8]–[9] that should have been detected by echolocation and avoided. Furthermore, many of these collisions involve illuminated objects that should have been detected by vision. Optomotor response tests indicate that the visual capabilities of insectivorous bats vary considerably, from species with modest acuity (e.g., *Myotis lucifugus*; [10]) to those with high acuity (e.g., *Macrotus californicus*; [11]). Visual sensitivity is generally optimal at conditions of low ambient light, such as dusk or dawn, and declines as light levels approach daylight [12]–[13], although there is species-specific variation in light tolerances [14]. The eyes of bats are optically adapted for long-distance use, where visual detection ranges exceed echolocation ranges [15]. The short-range visual capabilities of free-flying bats for orientation are largely unknown.

Determining how bats integrate visual and acoustic information is a key challenge in the sensory ecology of bats [16], as bats can attend to one or both senses, depending on context [17]. In situations where sufficient light enables both visual and acoustic orientation, conflicting information between the two modalities can result in captive bats preferentially attending to visual cues. For example, blindfolded Indiana bats (*Myotis sodalis*) released in a room during the day collided significantly less often with windows than non-blindfolded individuals [18]. Even though echolocation presumably presented windows as hard surfaces, non-blindfolded individuals repeatedly collided with the glass within one trial, suggesting they had not seen the windows and were using visual cues over conflicting auditory ones, or that the smooth glass surfaces were vertical echoacoustic mirrors and perceived as open flyways ([18], B.M. Siemers personal communication). Similarly, gray sac-winged bats (*Balantiopteryx plicata*) released individually into a mesh enclosure collided with the walls and ceiling more often in the day than the night [19]. Although these bats produced echolocation calls that should have enabled acoustic detection of the mesh, frequent collisions in the daylight support the precedence of visual cues over contradicting auditory cues. Little brown bats (*Myotis lucifugus*) flying through a laboratory obstacle course at different light intensities increased contact with obstacles when light intensities increased from dim to bright conditions [13]. Collectively, these data on captive bats demonstrate orientation using visual cues, and suggest that high levels of light affect orientation abilities, resulting in increased rates of collision. None of these experiments, however, manipulated the visibility of the objects with which the bats collided.

Free-flying bats may also attend to visual modalities preferentially under bright light conditions. McGuire and Fenton [20] recorded the frequency of collisions by free-flying *M. lucifugus* with a large trailer. They attracted bats with live conspecific distress calls, and observed that when lights were turned on, significantly more collisions occurred. They hypothesized that distress calls might compound the effects of light by acoustically distracting bats. Distress calls might also distract bats when vision is not involved.

We tested predictions arising from the hypothesis that free-flying bats are orienting by vision when they collide with some obstacles. We varied light intensities and the visibilities of obstacles. We predicted that if bats use vision to orient when visual cues are available, then the probability of collisions with obstacles would (1) be inversely proportional to the visibility of the obstacle in the light, and (2) be proportional to light intensity. We also predicted that (3) if distress calls act as acoustic distractions, bats would collide more frequently with obstacles, and that (4) compared to bats that avoided obstacles, the patterns of echolocation calls would differ in bats that collided with obstacles. Bats alter their echolocation calls in response to their immediate environment and typically decrease their call duration and interpulse interval when approaching objects compared to a uniform call pattern when no objects are present [21]–[22]. Therefore we expected that bats that collided with obstacles were orienting by vision (and not attending to echolocation), and would subsequently produce longer echolocation calls (with low variance) and longer interpulse intervals (with low variance) than bats avoiding collisions. We did not examine frequency parameters of individual calls because they are less reliable indicators of obstacle detection than the timing of pulse production.

## Methods

### Study Area and Subjects

We conducted our experiment from 6 to 30 August 2008 near an abandoned mine in Renfrew County, Ontario, that serves as a swarming and hibernation site (see [23]–[24] for details). Most bats there were *M. lucifugus* (98% of 1387 bats caught in traps; A. Adams personal communication) and both sub-adults and adults were present. Swarming begins in August, when bats of both sexes congregate at sites that will later serve as hibernacula, apparently to assess the site's suitability for hibernation, and later to mate and commence daytime torpor [23], [25]–[26]. The nightly arrival and departure of new bats during swarming [23], [27] reduced the risk of habituation and pseudoreplication. Accordingly, we considered each bat to be naïve to the experimental procedure, and did not capture or mark bats to reduce abnormal flight behaviour induced by the stress of being captured. Experimental procedures were approved by an Animal Use Protocol from the University of Western Ontario Council on Animal Care (2008-003-04) and a Wildlife Scientific Collector's permit from the Ontario Ministry of Natural Resources (1045694).

### Obstacle and Experimental Design

We used an obstacle made of plastic strips to assess the role of vision in *M. lucifugus*. To control for reliance on echolocation, we selected three visually distinct fabrics (opaque tablecloth, transparent tablecloth, and reflective emergency blanket). We tested their sound reflection properties under controlled laboratory conditions by broadcasting (ScanSpeak Ultrasonic speaker, Avisoft Bioacoustics, Berlin, Germany) synthetic echolocation calls (30–130 kHz) at constant intensity at the fabrics at a 90° angle, thereby maximizing echo-intensity. We conducted five trials for each fabric and for the no fabric control (where echoes returned from the laboratory walls). We used the minimum distance between the speaker/microphone and the fabrics to prevent the returning echoes from overlapping with outgoing calls. We used an ultrasonic condenser microphone (116/CM16, Avisoft Bioacoustics, Berlin, Germany) to record returning echoes and assess echo strength (a measurement of the intensity of the returning echoes; relative dB). All fabrics returned echoes of at least 46 dB, which were significantly greater than our no fabric control (ANOVA: F_3, 128_ = 1739.98 *p*<0.0001, Tukey's *post* hoc). Though there were differences in echo strengths between the fabrics, all fabrics were within the range of detection by *M. lucifugus* [28] and therefore presumed to be acoustically conspicuous to the bats.

We performed field experiments along a 25 m long by 3 m wide track that served as a flight corridor for the bats. We placed a 2.9 m wide by 2.6 m high frame across the flight corridor, and tightly secured 0.11 m wide fabric strips vertically across the frame, one fabric type at a time. We set gaps of 0.3 m between strips to ensure that most *M. lucifugus* (average wingspan 0.24 m; [29]) could maneuver through the inter-fabric space with no contact [30]–[31]. We secured a single condenser microphone (116/CM16, Avisoft Bioacoustics, Berlin, Germany) to the top of the frame, and angled it downwards at 45° in the direction of most approaching bats (herein defined as bats flying towards the obstacle). Recordings were made at a sampling rate of 250 kHz and a resolution of 8 bits.

We manipulated three variables: light intensity (dark, dim, and bright), fabric visibility (opaque, transparent, reflective) and the presence of distress calls (present/absent) for a total of eighteen treatment combinations. Each trial lasted 5 min, during which we recorded echolocation calls and video recorded the obstacle using two night-sensitive video cameras (model PV-GS35, Panasonic Canada Inc., Mississauga, Canada) and infrared lights (CCTV 48 IR LED Model S-8030, Scene Electronics, Shantou, China). Bats that came close to the obstacle and sharply turned away were not included in our study because of microphone and video detection constraints. Trials began at least 3 h after sunset to ensure no residual daylight, and terminated when bat activity levels tapered off (usually around 2 AM). We tested as many treatment combinations as possible during this period, randomizing the order. We did not conduct any trials in rain or on windy nights (>2 m/s).

We tested three light intensities- dark, dim, and bright- with approximate intensities of 0, 5, and 340 lux respectively. We used a luxmeter to measure the light intensity in the middle and outside corners of the obstacle for each trial (Mastech LX1010B, Mastech, Kowloon City, China). The dark condition was ambient light. For dim and bright conditions, we directed one spotlight at the obstacle from 3 m distance, angled upwards 45° from the ground. The lights faced the obstacle in the direction of most approaching bats to increase the contrast of the fabrics, and to prevent shining the lights directly into the bats' eyes as they approached.

We used distress calls produced by ten adult *M. lucifugus* confined in a Hitchcock holding cage (cylindrical metal cage made from wire mesh) to attract bats to the obstacle. We used live distress calls because they elicit greater responses from free-flying bats than playbacks of distress calls [32]. We collected new bats every 30 min to avoid restricting foraging opportunities for these individuals and to ensure continuous distress calls throughout trials. During a distress call trial, we centered the cage with confined bats 1.5 m behind the obstacle. During a silent trial, we placed an empty cage 1.5 m behind the obstacle as a control [23], [33], moved the confined bats 35 m away, and waited 2 min before starting the trial to minimize residual activity in the experimental area elicited by the trapped bats [34].

### Obstacle Avoidance Analysis and Statistics

We recorded time-marked observations of approaches by bats during trials and classified each encounter with the obstacle as a pass or collision. We analyzed videos using MotionDV Studios (Panasonic version 5.3E, Matsushita Electric Industrial Co., Kadoma, Japan), and categorized any contact with the fabrics as a collision. These were unmistakable events on the video record and could be recognized by the sound of fabric crinkling, the fabric moving, or the abrupt wing motions of the bat upon collision.

We used ANOVA (SPSS version 17.0, SPSS Inc.) to determine the effects of our manipulated variables (lights, fabrics, and distress calls) on overall activity (passes and collisions). The overall activity data were not normally distributed, so we used their natural logarithms to meet assumptions of normality (Kolmogorov-Smirnov test).

We used a logistic regression model (SAS version 9.1, SAS Institute Inc.) to test for the effects of light (dark, dim, bright), obstacle visibility (transparent, opaque, reflective), distress calls (present, absent), date, and time (minutes after sunset) on the probability of bats colliding with obstacles in each trial. Categorical variables (light, obstacle visibility, and distress calls) were coded with dummy variables, and continuous variables (date and time) were centered on their means to reduce the risks of collinearity. We used a backwards elimination procedure with a likelihood ratio test to select the final logistic regression model. There were significant interaction terms between continuous and categorical variables, so we generated parameter estimates of the categorical variables by level (i.e., the light variable was broken down into bright, dim, and dark). We used parameter estimates to present the effects of each treatment on the probability of bats colliding with obstacles. We calculated odds ratios (comparisons of the relative likelihood of two events occurring) to contrast the probabilities of collisions among light levels and among obstacle types.

### Echolocation Analysis and Statistics

We used callViewer software (version 16; [35]) for all sound analysis. For each of the eighteen treatment groups, we selected five sequences of six consecutive echolocation calls from files recorded throughout the study month. It was not possible to determine the flight paths of the bats that produced the calls. We measured the parameters of the call of greatest amplitude and the five preceding calls for each sequence, assuming that increasing amplitude indicated decreasing distance from the microphone. All analyzed calls in the sequence were at least 10 dB above background noise, and between 10% and 100% of maximum resolution. We removed harmonics and echoes with the callViewer signal to noise ratio filter. We assigned calls to a pass or collision category to the best of our ability by confirming that no other bats were present on the video record at the time of the encounter with the obstacle, and by ensuring that there was at least a 3 s gap between the sequence being analyzed and the previous and next sequence of calls. We affiliated sequences of calls with a date and a pass or collision classification only after we processed all calls in callViewer to prevent biasing the data.

We measured the call duration and interpulse interval (IPI; time between the start of one call and the start of the next call). For each sequence of calls, we calculated the mean and variance of the duration and IPI. Although we could not determine if the calls were produced as the bats approached or moved away from the obstacle, we predicted the variance data would increase, regardless of flight path, for bats that acoustically detected the obstacle [22].

We examined the effects and interactions of lights, fabrics, distress calls, date, time (minutes after sunset), and passes or collision on the duration, duration variance, IPI, and IPI variance using ANCOVA (SPSS version 17.0, SPSS Inc.). The variance data were not normally distributed, so we used their natural logarithms to meet assumptions of normality (Kolmogorov-Smirnov test).

## Results

### Activity level

**Figure 1:**
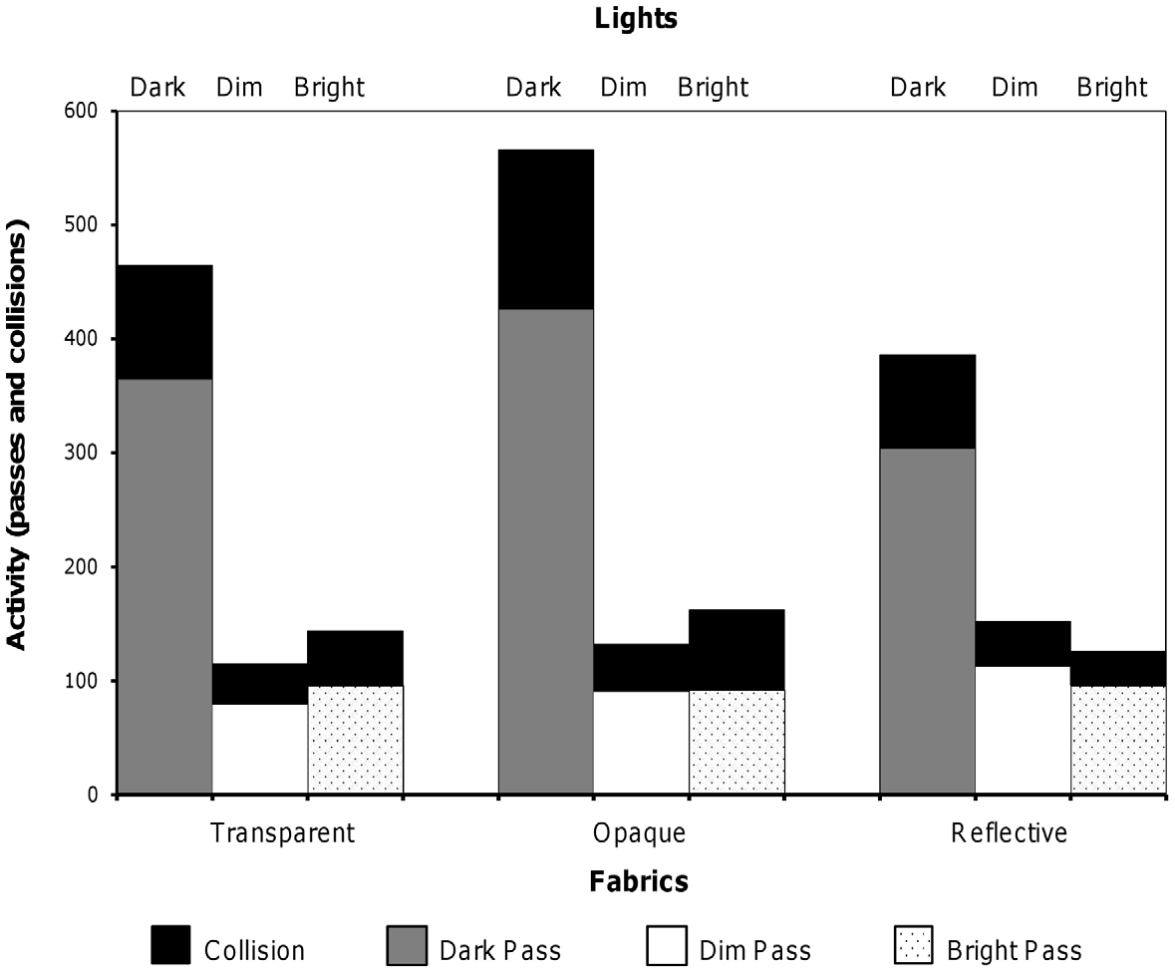
Total approaches to the obstacle. The combined passes (dark: grey, dim: white, bright: dotted) and collisions (black) of bats with the obstacle for the three fabric types (transparent: n = 74 trials, opaque: n = 73 trials, reflective: n = 79 trials) for the three light levels (dark: n = 99 trials, dim: n = 61 trials, bright: n = 66 trials).

Over 226 trials, we observed a total of 2248 approaches to the obstacle by bats, of which 26% resulted in collisions. Total activity (passes and collisions) per trial was affected by lights (*F*_2, 220_ = 25.26 *p*<0.0001; Figure 1) and distress calls (*F*_1, 220_ = 90.97 *p*<0.0001), but not by fabrics (*F*_2, 220_ = 1.57 *p* = 0.21). There was more activity in the dark than in dim or bright lights, suggesting bats avoided light, and no difference between dim and bright lights (Tukey's *post hoc*; Figure 1). There was more activity with distress calls than without (trials with distress calls: mean ± S.E.  = 13.07±1.23, trials without distress calls: mean ± S.E.  = 5.40±0.66).

### Obstacle Avoidance

Light intensity and obstacle visibility affected the probability of collision, and both variables were related to date (light x date: Wald χ^2^ = 45.35, 2 d.f., *p*<0.0001; fabric x date: Wald χ^2^ = 8.20, 2 d.f., *p* = 0.017). Parameter estimates indicated that all fabric and light levels interacted significantly with date except for the opaque fabric (Table 1).

**Table 1:** Parameter estimates.

Variable	Parameter Estimate	Standard Error	Wald χ^2^	p-value
Intercept	−1.04	0.14	57.21	<0.0001
Opaque	0.21	0.16	1.56	0.211
Reflective	−0.27	0.18	2.20	0.138
Dim	0.19	0.19	1.08	0.298
Bright	0.28	0.18	2.54	0.111
Date	0.10	0.02	25.27	<0.0001
Date x Opaque	0.01	0.02	0.15	0.697
Date x Reflective	0.07	0.03	7.03	0.008
Date x Dim	−0.12	0.03	17.65	<0.0001
Date x Bright	−0.14	0.02	39.61	<0.0001

d.f.  = 1.

Parameter estimates from the logistic regression model of all significant variables on the probability of bats colliding with obstacles. These parameter estimates show significance levels of the main effects (fabric and light), and interaction terms with date. The effects of transparent and dark treatments are included in the intercept term by SAS.

**Figure 2:**
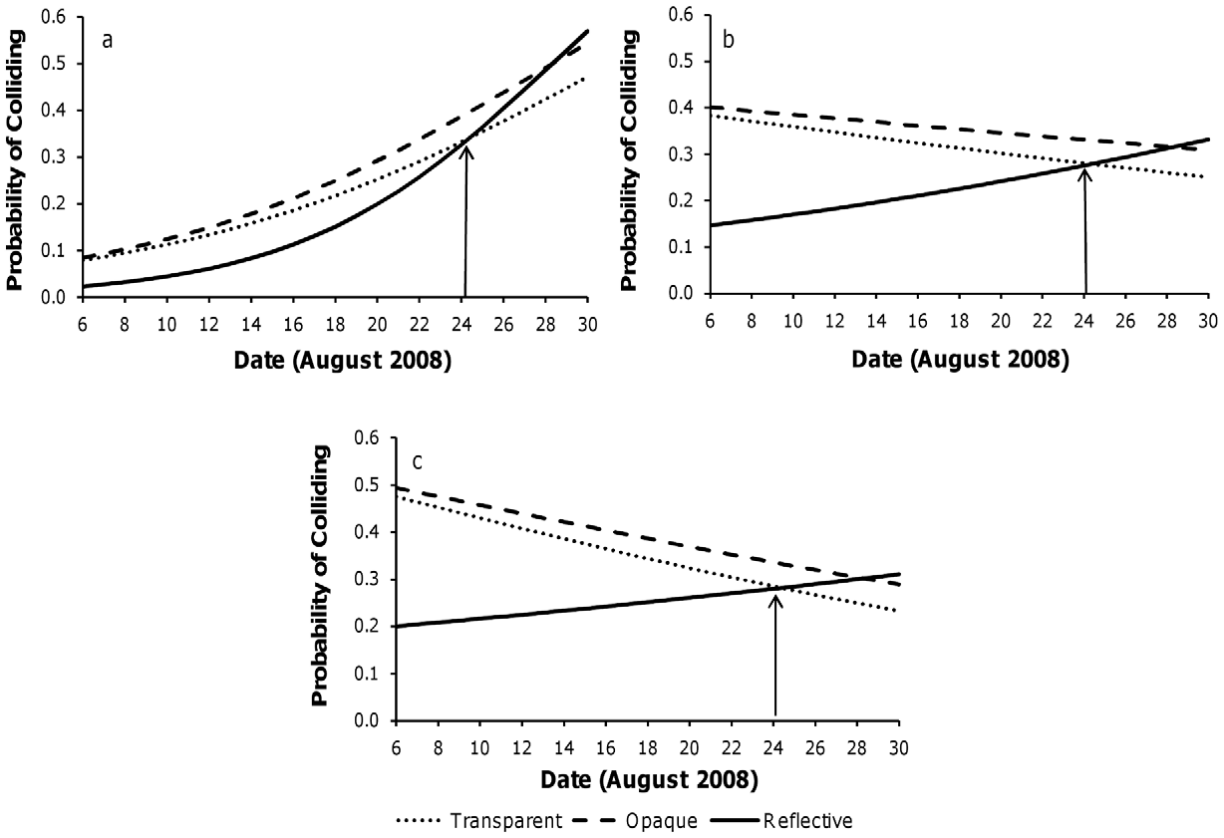
Probabilities of colliding as a function of fabric type and date. The probabilities of bats colliding with obstacles for each fabric type, with light levels fixed for (a) dark (n = 99 trials), (b) dim (n = 61 trials), and (c) bright (n = 66 trials) conditions. The arrow marks the cross-over point in the month where there is a change in the probability of colliding with obstacles.

With light levels fixed, the probability of bats colliding with the opaque and transparent fabrics were similar, regardless of date (date x (opaque-transparent contrast): Wald χ^2^ = 0.15, 1 d.f., *p* = 0.679; Figure 2, Table 2). Bats initially collided less often with the reflective fabric than the opaque/transparent fabrics in the light, but this pattern changed from August 24^th^ to the end of the month, where bats appeared to collide equally with all fabrics (date x (reflective-transparent contrast): Wald χ^2^ = 7.03, 1 d.f., *p* = 0.008, date x (opaque-reflective contrast): Wald χ^2^ = 5.97, 1 d.f., *p* = 0.015; Figure 2, Table 2). We reanalyzed the data only considering dark trials, and found that in the absence of artificial light, bats collided equally with all fabrics (Wald χ^2^ = 4.86, 2 d.f., *p* = 0.088; Figure 2a).

**Table 2:** Odds ratios.

Odds Ratio Contrasts	Odds Ratio	Standard Error	Wald χ^2^	p-value
Opaque to Transparent	1.22	0.20	1.56	0.211
Reflective to Transparent	0.76	0.14	2.20	0.138
Opaque to Reflective	1.61	0.28	7.50	0.006
Dim to Dark	1.21	0.22	1.08	0.298
Bright to Dark	1.33	0.24	2.55	0.111
Dim to Bright	0.91	0.20	0.17	0.679
Date	1.10	0.02	25.27	<0.0001
Date x (Opaque to Transparent)	1.01	0.02	0.15	0.697
Date x (Reflective to Transparent)	1.07	0.03	7.03	0.008
Date x (Opaque to Reflective)	0.94	0.02	5.97	0.015
Date x (Dim to Dark)	0.88	0.03	17.65	<0.0001
Date x (Bright to Dark)	0.87	0.02	39.61	<0.0001
Date x (Dim to Bright)	1.02	0.03	0.40	0.526

d.f.  = 1.

Odds ratios and significance levels of the main effects and interaction term contrasts on the probabilities of bats colliding with obstacles. Each contrast explains the likelihood of collision under one condition relative to a second condition. Main effects (fabric or light) odds ratios are fixed at the mean month date (derived considering the number of trials per night), while the interaction term (date) odds ratios show the effect of a 1 day increase in date on the main effects odds ratios.

**Figure 3:**
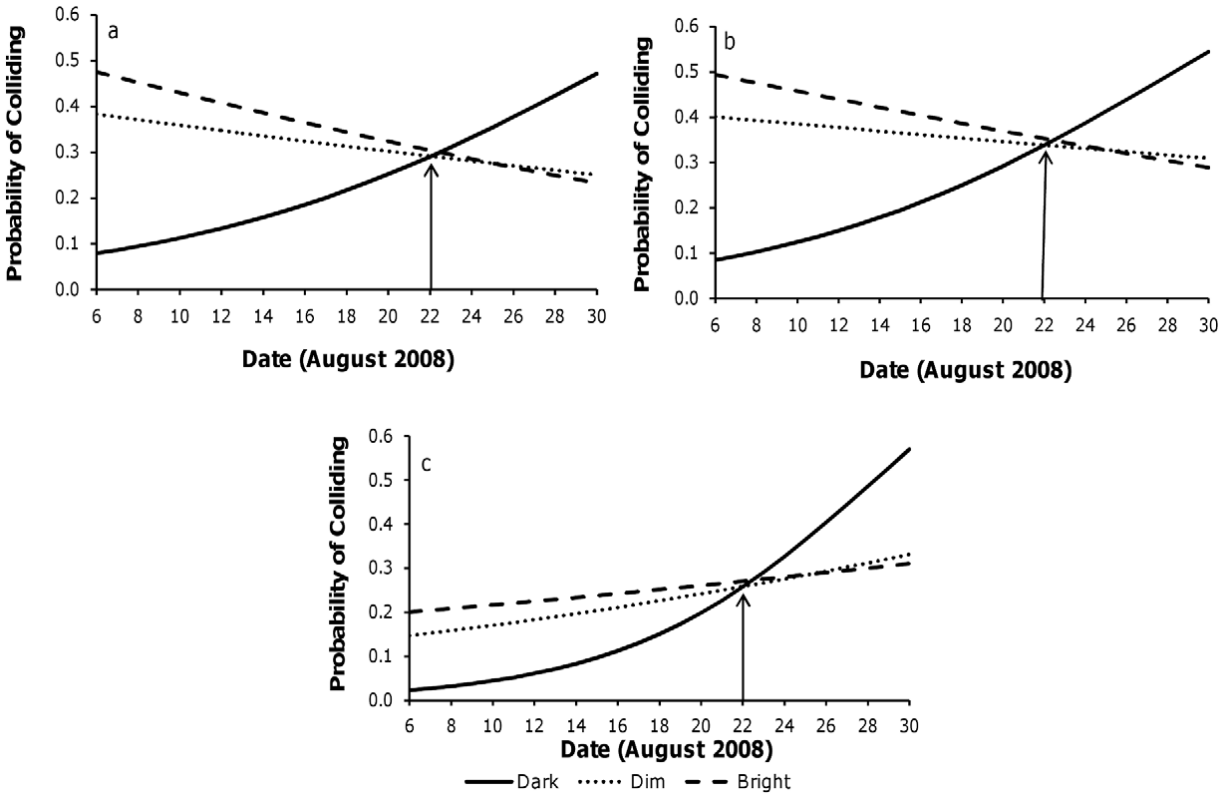
Probabilities of colliding as a function of light level and date. The probabilities of bats colliding with obstacles for each light level, with fabric types fixed for (a) transparent (n = 74 trials), (b) opaque (n = 73 trials), and (c) reflective (n = 79 trials) conditions. The arrow marks the cross-over point in the month where there is a change in the probability of colliding with obstacles.

With fabric types fixed, collision probabilities were similar between dim and bright lights, regardless of date (date x (dim-bright contrast): Wald χ^2^ = 0.40, 1 d.f., *p* = 0.526; Figure 3, Table 2). Initially, bats collided less often in the dark than the light, but this behaviour reversed around August 22^nd^, where bats collided most often in the dark for the remainder of the month (date x (dim-dark contrast): Wald χ^2^ = 17.65, 1 d.f., *p*<0.0001, date x (bright-dark contrast): Wald χ^2^ = 39.61, 1 d.f., *p*<0.0001; Figure 3, Table 2).

### Echolocation

We found no changes in the duration, IPI, or IPI variance in response to any predictor variables (lights, fabrics, distress calls, date, time, and passes or collisions). However, duration variance decreased in the presence of distress calls (*F*_1, 81_ = 5.09 *p* = 0.027; with distress calls: n = 45, mean (ms) ± S.E.  = 0.16±0.03; without distress calls: n = 45, mean (ms) ± S.E.  = 0.27±0.05). Duration variance was not affected by any other predictor variables.

## Discussion

Echolocation appears to be the primary sensory modality in the orientation of vespertilionid bats [16], however, under our experimental conditions bats appeared to use vision when visual cues were available. We discount the use of spatial memory (orienting in a familiar area by relying on memories of the environment and its space) or acoustic landmarking (orienting in a familiar area by relying on acoustic cues to recall features of space) as bats should have collided equally with obstacles in all conditions if they oriented by these strategies.

We additionally discount the possibility that our bats used only echolocation under lit conditions. Although bats can use echolocation and vision simultaneously (the two are not mutually exclusive), many produce echolocation calls even when orienting by vision [19], [36]–[38]. If the bats in our study relied exclusively on acoustic cues, then the probabilities of colliding with one fabric on one date should not have varied, as the only change was light level (i.e. there should have been equal probabilities of colliding with the opaque fabric in the bright and dark conditions on Aug 9^th^ if the bats used only echolocation). Similarly, Masterson and Ellins [39] concluded that the response of *M. lucifugus* to brightness cues alone supports some reliance on vision. We cannot discount the possibility that our study animals acoustically detected the obstacles and ignored the cues. Unlike previous anecdotal reports or studies with captive bats in unnatural settings [13], [18]–[19], [36], our study supports a relationship between the availability of visual cues and the use of vision by free-flying *M. lucifugus* for orientation.

Free-flying bats might collide with stationary objects because of reliance on vision when visual cues are available, despite limited visual capabilities in brighter lights. Bats are nocturnal and are generally exposed to limited light. Accordingly their optical system has evolved and the densely packed rods in the retinas of bats converge on a few ganglion cells to provide good light-gathering capabilities, but these develop at the expense of visual acuity by reducing the eye's ability to separate distinct retinal image points [40]–[42]. *Myotis lucifugus* has comparatively poor visual acuity compared to other bats [10], possibly explaining the occurrences of collisions in the light. We do not consider collisions with moving objects, such as wind turbines, because bats are better at avoiding moving objects than stationary ones [30], [43].

Bats did not behave differently in bright and dim light treatments, possibly because both conditions were overwhelming compared to dark treatments. During laboratory studies on vision, bats can acclimate to fixed unnatural ambient lights before performing tasks [12]–[13], [44]–[45]. In our field experiment we could not adjust ambient light levels more than 3 m beyond the obstacle. Accordingly, bats flying in relative ambient darkness (<1 lux) were suddenly exposed to comparatively bright lights as they approached the high contrast fabrics, analogous to turning on a light in the night and becoming momentarily disoriented and blinded. We suggest the sudden change in ambient light levels may explain why the responses of free-flying bats to dim and bright lights in our study differ from laboratory observations [13]. Recognizing the limitations of laboratory studies on vision in their application to field techniques is essential when trying to accurately determine orientation mechanisms [37].

The observed changes in mid to late August (Figures 2 and 3) correspond with behavioural, hormonal, and physiological changes occurring in bats during swarming [26], [33], [46]–[47]. There are two phases of swarming, which are distinguished by behaviour and activity patterns. The first phase begins in late July at our field site, and consists of extensive foraging to deposit fat stores for hibernation, and potential assessment of the site's suitability for hibernation [23], [48]. The second phase of swarming is marked by the onset of promiscuous mating [25], [33], daytime torpor bouts [26], and changes in nutrient intakes [26], all commencing in mid to late August at our study site [23], [26], [33]. These changes all occur at the same time we observed differences in the order of the probability of bats colliding with obstacles. Although behavioural changes occurred gradually over August, the correspondence of the dates suggests that bats' visual attentiveness in flight could be synchronized with the transition between the two phases of swarming. Future research into the integration of behavioural and physiological changes associated with the two phases of swarming is warranted.

Free-flying bats respond to distress calls by increasing activity in the vicinity of the calling bats [32], [49]. While we observed the same trend in activity patterns, our data do not support the hypothesis that free-flying bats were acoustically distracted, as the probabilities of collision did not change in the presence of distress calls.

The lack of detected changes in echolocation calls parameters between bats that passed through or collided with obstacles suggest that vision was a prominent modality for orientation. We were unable, however, to localize bats in space and time, and our microphone could have detected calls from bats that were sufficiently far away from the obstacle that they had not yet started to acoustically respond. Future field studies synchronizing acoustic and visual data during obstacle avoidance tasks are necessary.

Our study is a comprehensive examination of the role of visual cues on the collisions of free-flying *M. lucifugus* with obstacles. Past work on the use of vision for orientation in echolocating bats has yielded conflicting results. Captive bats may orient towards light sources to escape [18]–[19], [37], [50], or may refuse to fly at all [18]. Therefore obstacle avoidance experiments with captive bats must be approached with caution when predicting the behaviour of free-flying bats. Our study suggests that vision plays a larger role in the short-range orientation behaviour of free-flying bats than previously recognized, and we advocate a need for more controlled experiments in natural settings when assessing sensory modality integration in bats.
